# Application of Holistic Liquid Chromatography-High Resolution Mass Spectrometry Based Urinary Metabolomics for Prostate Cancer Detection and Biomarker Discovery

**DOI:** 10.1371/journal.pone.0065880

**Published:** 2013-06-18

**Authors:** Tong Zhang, David G. Watson, Lijie Wang, Muhammad Abbas, Laura Murdoch, Lisa Bashford, Imran Ahmad, Nga-Yee Lam, Anthony C. F. Ng, Hing Y. Leung

**Affiliations:** 1 Strathclyde Institute of Pharmacy and Biomedical Sciences, Glasgow, Scotland, United Kingdom; 2 Department of Urology, Gartnavel General Hospital, Glasgow, Scotland, United Kingdom; 3 The Beatson Institute for Cancer Research, Glasgow, Scotland, United Kingdom; 4 Glasgow Clinical Research Facility, Glasgow, Scotland, United Kingdom; 5 Department of Urology, Chinese University of Hong Kong, Hong Kong; Florida International University, United States of America

## Abstract

Human exhibit wide variations in their metabolic profiles because of differences in genetic factors, diet and lifestyle. Therefore in order to detect metabolic differences between individuals robust analytical methods are required. A protocol was produced based on the use of Liquid Chromatography- High Resolution Mass Spectrometry (LC-HRMS) in combination with orthogonal Hydrophilic Interaction (HILIC) and Reversed Phase (RP) liquid chromatography methods for the analysis of the urinary metabolome, which was then evaluated as a diagnostic tool for prostate cancer (a common but highly heterogeneous condition). The LC-HRMS method was found to be robust and exhibited excellent repeatability for retention times (<±1%), and mass accuracy (<±1 ppm). Based on normalised data (against creatinine levels, osmolality or MS total useful signals/MSTUS) coupled with supervised multivariate analysis using Orthogonal Partial Least Square-Discriminant Analysis (OPLS-DA), we were able to discriminate urine samples from men with or without prostate cancer with R2Y(cum) >0.9. In addition, using the receiver operator characteristics (ROC) test, the area under curve (AUC) for the combination of the four best characterised biomarker compounds was 0.896. The four biomarker compounds were also found to differ significantly (P<0.05) between an independent patient cohort and controls. This is the first time such a rigorous test has been applied to this type of model. If validated, the established protocol provides a robust approach with a potentially wide application to metabolite profiling of human biofluids in health and disease.

## Introduction

Prostate cancer is the most prevalent cancer in the male population in Western countries. Prostate cancer is highly heterogeneous with highly variable clinical outcomes: indolent disease tends not to progress even over many years while aggressive (high grade) disease often progresses quickly to result in metastases which inevitably result in premature death. In addition, there is a significant limitation in specificity with the current practice using serum prostate specific antigen (PSA) measurement as a diagnostic tool. Hence, there is an urgent need for better diagnostic and prognostic tests for prostate cancer.

Evolving evidence points to the input of highly versatile metabolic pathways in fuelling carcinogenesis [1] thus detailed analysis of the tumour-associated metabolome may reveal novel biomarkers [2], [3]. Analysis of urine, plasma and/or tissue samples can be performed with Nuclear Magnetic Resonance (NMR) spectroscopy or/and Mass Spectrometry (MS) combined with separation techniques such as Liquid Chromatography (LC) and/or Gas Chromatography (GC). Sreekumar *et al*. carried out untargeted metabolomic profiling for prostate cancer using LC/GC-MS across three different types of clinical samples (urine, plasma and tissue). Over 1,000 features were detected across the samples analysed [4]. A six-metabolite profile was suggested to signify high risk of cancer progression and additional isotope dilution GC-MS analysis identified sarcosine as a potential urinary biomarker for aggressive prostate cancer. Disappointingly, sarcosine as a (urinary or plasma) biomarker in prostate cancer has not been supported by several independent studies subsequently [5]–[15]. In addition, only a handful of these studies have attempted to perform untargeted metabolite profiling [6], [16], [17]. In these subsequent studies, the use of GC-MS or differential mobility analysis (DMA)-MS provided even more limited coverage of metabolites when compared to the study by Sreekumar *et al* [4]. Hence, there is a real need to develop a robust methodological and analytical platform to facilitate future efforts in global profiling of the metabolome in prostate cancer biomarker discovery [18].

Urine has some advantages for metabolomics studies since it has a high abundance of metabolites, low content of protein, is collected non-invasively and requires minimal sample preparation prior to analysis. Recently, application of high resolution (HR) MS based urinary metabolomics has gained popularity in the study of cancer diagnosis and biomarker discovery, especially in combination with diverse LC techniques. HRMS shows higher coverage of the metabolite profile than other methods and the ability to identify potential biomarker compounds [19]–[24]. The introduction of Hydrophilic Interaction Liquid Chromatography (HILIC), where the retention mechanism is orthogonal to that of Reversed Phase (RP) Liquid Chromatography, offers a suitable separation platform for the many highly polar metabolites in urine [25]. In a recent study of bladder and kidney cancer biomarkers [22], combined multivariate analysis (MVA) and Orthogonal Partial Least Square-Discriminant Analysis (OPLS-DA) of pooled data from RP and HILIC LC-HRMS showed 100% accuracy in segregating cancer and healthy subjects correctly.

Data normalisation is considered an essential but unstandardised step in human urinary analysis [26]. Principal Component Analysis (PCA) score plots can be rendered completely different simply depending on the normalisation method used, and the value of candidate biomarkers can be invalidated by choosing a “wrong” internal component as the reference. The signal/level of creatinine and total ion current (TIC) are commonly used as normalisation factors in LC-HRMS based urinary metabolite profiling studies [4], [20], [22], [24]. Warrack *et al* introduced a new normalisation strategy based on the MS Total Useful Signals (MSTUS) which had encouraging correlation to the data based on normalisation to urinary osmolality and recommended using at least two different normalisation methods to ensure statistically significant changes in metabolite profile [27].

A protocol using a combination of GC-MS and LC-MS to carry out metabolic profiling of plasma and serum was recently described [28]. Unlike urine it is not necessary to normalise the data for blood derived-samples in metabolomics studies. Although comprehensive protocols using GC-MS and LC-MS to profile the urinary metabolome have also been reported [29], [30] none of them have discussed or compared normalisation methods to any great extent. In addition, the metabolite coverage by GC-MS is necessarily limited to volatile components. The combination of two orthogonal LC methods for metabolomic profiling has only been applied during the period since the protocols described in references 28 and 30. Building on our earlier work [25], we have further optimised our methodology and analysis pipeline, and profiled urine samples from patients with prostate cancer and control urines by LC-HRMS using orthogonal separation methods. The effect of three different normalisation methods in data analysis was demonstrated. By using the results of clinical tests the discriminating ability of metabolomic profiling of urine in relation prostate cancer was evaluated by using both OPLS-DA models and specific biomarkers. The study was guided by the STAndards for the Reporting of Diagnostic accuracy (STARD) criteria [31] and the evaluation checklist can be found in (File S1).

## Materials and Methods

### Chemicals and materials

HPLC grade acetonitrile (ACN) was purchased from Fisher Scientific, UK. HPLC grade water was produced by a Direct-Q 3 Ultrapure Water System from Millipore, UK. AnalaR grade formic acid (98%) was obtained from BDH-Merck, UK. Ammonium carbonate and ammonium acetate were purchased from Sigma-Aldrich, UK.

### Sample collection

All samples studied were obtained with appropriate written consent from patients. The collection of samples was approved by the institutional ethics review board (Joint The Chinese University of Hong Kong - New Territories East Cluster Clinical Research Ethics Committee). Details on patient-related clinical information including prostate cancer parameters are described in Table 1.

**Table 1:** Clinicopathological characteristics of the tumor patients.

PSA at collection ng/ml	DRE at collection	GS1	GS2	T	N	M	localized/Locally advanced/metatstatic dx
4.6	2a	3	3	2a	0	0	1
12.3	1	3	3	1c	0	0	1
10.1	1	4	3	1c	0	0	1
39.5	2a	4	3	2a	0	0	1
9	1	3	3	1c	0	0	1
8.8	1	3	3	1c	0	0	1
17.7	1	4	4	1c	0	0	1
6.2	1	4	4	1c	0	0	1
9.6	2c	3	5	2c	0	0	1
11.4	1	3	4	1c	0	0	1
1.48	2c	3	3	3b	0	0	2
8.7	2b	4	4	2b	0	0	1
29.5	2b	3	4	2b	0	0	1
51.6	3	3	3	3	0	0	2
63.3	3	4	5	3b	1	0	2
6.6	1	3	3	1c	0	0	1
23.6	2	4	4	3b	1	1	3
12.2	1	3	3	1c	0	0	1
9.4	1	3	4	1c	0	0	1
11.8	1	3	5	1c	0	0	1
52.2	4	4	5	4	0	0	2
35.5	3	3	5	3b	0	0	2
38	2a	4	3	2a	0	0	1
11.4	2a	3	4	2a	0	0	1
54.8	2c	3	4	3b	1	0	2
44.4	2b	4	3	2b	0	0	1
8.2	1	3	3	1c	0	0	2
413	3	5	4	3	0	1	3
32.1	3	3	4	3a	0	0	2
25.5	2b	3	4	3b	0	0	2

**GS = Gleason Score T = T stages N = N stages M = M stages.**

### Sample preparation

The urine samples were stored at −30°C and thawed at room temperature before preparation for LC-MS analysis. For analysis using HILIC conditions, 200 µl of urine was thoroughly mixed with 800 µl of acetonitrile, followed by centrifugation at 3000 revolutions per minute (RPM) for 5 minutes; 800 µl of supernatant was then transferred to a LC vial. For the RP conditions 200 µl of urine was diluted with 800 µl of water in a LC vial. The pooled sample was prepared by gathering 100 µl of urine from each sample which was then treated as above.

### Measurement of creatinine and osmolality

50 µl of diluted samples and prepared creatinine standard solutions were thoroughly mixed with 100 µl of creatinine detection reagent (Enzo Life Sciences, UK) in a 96-well plate and the absorbance was read at 490 nm by using a Spectra Max M5 from Molecular Devices. The concentrations of creatinine in the test samples were calculated using a 7-point calibration curve in which each point was measured in duplicate. After calibrating with standard solutions (Vitech Scientific Ltd., UK), freezing-point depression measurement for each sample was performed with an osmometer (Advanced Micro, Model 3300) in order to determine the osmolality.

### LC-MS data acquisition

Samples were randomly placed in the autosampler tray and the LC-MS experiment was performed on an Accela 600 HPLC system combined with an Exactive (Orbitrap) mass spectrometer from Thermo Fisher Scientific (Bremen, Germany). A ZIC-pHILIC column (150×4.6 mm, 5 µm) and also an ACE C18-AR column (150×4.6 mm, 5 µm) (HiChrom, Reading UK) were employed for HILIC and RP separations respectively. The mobile phases used in HILIC conditions were 20 mM ammonium carbonate buffer (pH 9.2) and pure ACN Under RP conditions 0.1% v/v formic acid in water and 0.1% v/v formic acid were used as the mobile phases. The HILIC and RP LC eluting gradient profiles and the MS parameter settings were described in our previous study [25]. The selective MS^2^ fragmentation of potential biomarkers was carried out by using Collision Induced Dissociation (CID) at 35 V using a Surveyor HPLC system combined with a LTQ-Orbitrap mass spectrometer from Thermo Fisher Scientific (Bremen, Germany).

### LC-MS data processing by MZMine 2.10 [32]

The raw data was split into a single ESI positive and negative data set, and also converted into mzML format by using ProteoWizard. The procedure and the settings of each step used in MZMine 2.10 are described in File S2. Here the strategy of using the data of pooled samples to filter out technical and unrelated biological variations from the data set was introduced. The five pooled samples were prepared along with test samples and they were measured as quality controls (QCs) periodically throughout the whole LC-MS experiment. One of them was injected three times and the rest were injected only once, therefore 7 sets of QC data were acquired to assess system stability and to generate a repeatability filter. The features not presenting in all 7 QC data sets were removed because they were either caused by the variation of the sample preparation or by the LC-MS system during the data acquisition. Based on this filtered peak list, the data for individual test samples were aligned and all features not matching with the pooled sample peak list were removed. Features within the QC data set are included only if they are observed in >75% of the test samples. Finally, the features with a relative standard deviation (RSD) for peak area less than 25% within the QCs were used for further data analysis.

### Normalisation

For the creatinine or osmolality normalisation method, the peak area of each feature in a sample was scaled to the creatinine concentration or osmolality of the sample. For MSTUS method under each combination of LC condition and ESI mode, the peak areas of all features which remained through the three filters were summed up and then were replaced by their percentages of the total value.

### Multivariate/statistical analysis

SIMCA-P 13 (Umetrics, Sweden) was used to carry out all MVA. Prior to PCA and OPLS-DA, the data were mean-centered and unit variance (UV) scaled. Pareto scaling was also tried but the models generated were dominated by a few features with large normalised values (e.g. creatinine and the dimer of urea). Thus, considering the fact that in searching for biomarkers all features are biologically equal and that most of the noise was removed by the filters, UV scaling was used. P-values were calculated by the two tail Student *t* test (Microsoft Office 2010). The ROC test was performed by IBM SPSS Statistics 21.

## Results and Discussion

### LC-HRMS method evaluation and validation

Untargeted metabolomic profiling based on two orthogonal LC separation methods was performed [33]: A ZIC-pHILIC column with a mobile phase pH at 9.2 for the HILIC method and an ACE C18-AR column with a mobile phase pH at 2.6 for the RP method, referred hereafter as “ZIC-pHILIC” and “RP” respectively. In order to examine the complementarity of these two orthogonal LC methods some standard metabolites were measured along with the samples under each LC condition. As shown in our previous study alanine and β-alanine can be separated under ZIC-pHILIC conditions [25]. Here a clear separation of the isomers sarcosine, which was previously suggested as prostate cancer marker, alanine and β-alanine was also achieved under ZIC-pHILIC conditions but they all eluted at the column dead volume under RP conditions (Figure S1 and S2 in File S3). The LC-HRMS raw data were processed by MZMine 2.10 as described in the Experimental section. Table 2 shows the number of features remaining after each filter and their percentages based on the originally detected features. The pooled sample repeatability filter effectively removed more than 75% of detected features, most of which originated from LC-HRMS system noise and background signals. The numbers of features were modestly decreased through the 25% missing filter, which removed biological compounds which were very variable within the samples. Through the peak area RSD filter features with large peak area variations can be removed. Such features are generally due to low MS responses or poor chromatographic peak shapes. Approximately 5,200 features in total survived these three filters and were pooled together as a holistic dataset for multivariate and statistical analysis.

**Table 2:** The numbers of remaining features and their percentages after each data filter.

LC conditions and ESI modes	Originally detected in all samples	After PS filter	After 25% missing filter	After 25% RSD filter (Final)
ZIC-pHILIC-Pos	7910 (100%)	1839 (23.25%)	1559 (19.71%)	1341 (16.95%)
ZIC-pHILIC-Neg	13254 (100%)	3327 (25.10%)	2875 (21.69%)	1676 (12.65%)
RP-Pos	6697 (100%)	1460 (21.80%)	1233 (18.41%)	1022 (15.26%)
RP-Neg	9264 (100%)	1691 (18.25%)	1416 (15.28%)	1209 (13.05%)

The stability of the LC-HRMS system was verified by comparing the accurate masses (±3 ppm) and retention times (±0.5 min) of compounds present in the urine against authentic standards: 42 and 51 features were matched to the standards under ZIC-pHILIC conditions in ESI positive and negative modes respectively, and 39 and 34 features were assigned under RP conditions. The system stability was monitored with respect to variation in retention time, peak area and mass accuracy of these identified metabolites in 7 QC samples under each LC-HRMS condition (File S3). After MZMine processing, most of the standard matched metabolites present in the samples showed extremely low RSDs for mass accuracy (<1 ppm) and retention time (<1%). Only few of them showed large variations for peak area (>25%) because of their low MS responses; these were excluded by the 25% RSD filter. Having developed the data filtering methodology these filters were then applied to the data from the 30 patient and 30 control samples. The features remaining after applying the filters were then further processed.

### Normalisation and multivariate/statistical analysis

**Figure 1:**
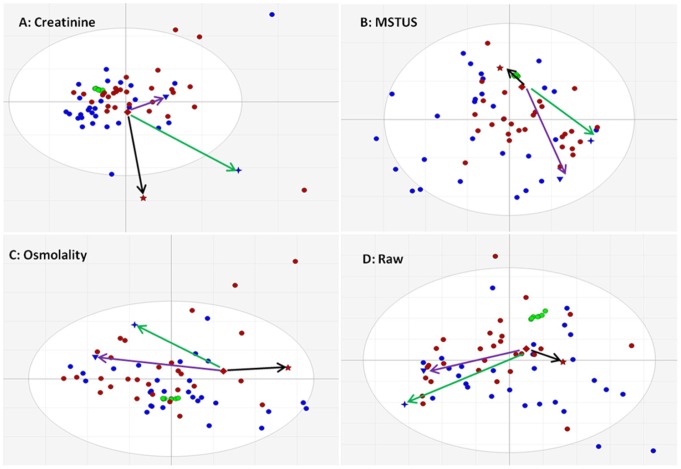
PCA score plots with different normalisation methods. Cancer subjects are labelled in red, controls in blue and QCs in green. The vector from diamond to 5-point star is labelled in black, to 4-point star in green and to inverted triangle in purple. (A–C) Normalisation to creatinine, MSTUS and osmolality respectively. (D) raw data without normalisation.

**Figure 2:**
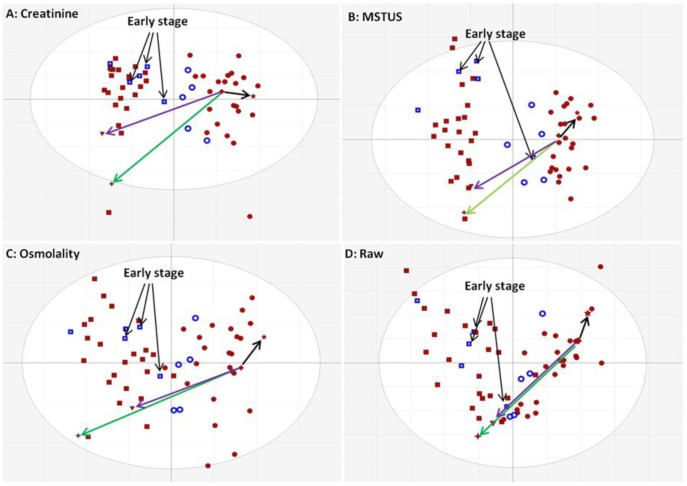
OPLS-DA score plot with different normalisation methods. Cancer subjects are shaped as squares and controls as circles. The training set is labelled in filled red and the test set in hollowed blue. The four selected subjects and the vectors are same shaped and coloured as Figure 1.

There is currently no consensus on normalisation method(s) in urine metabolomics studies. We applied three independent normalisation methods and the corresponding PCA score plots are shown in Figure 1. Given the diversity in life style between subjects, unsupervised MVA did not separate the samples from the cancer and control groups. In addition no similarity could be observed between the pattern structures which is reflected by the completely different directions and distances (vectors) based on one sample (shaped as diamond) referenced to three other samples (shaped as 4-point star, 5-point star and inverted triangle) in each pattern. The sum of the first and the second principal components is less than 30% for each model reflecting the poor correlation among the variables (features) in the dataset. Importantly, in all four PCA models, the 7 QC features (in green) are close each other as a cluster in the middle of the pattern, further confirming excellent system stability throughout. As a supervised MVA method, OPLS-DA was able to separate study groups according to predefined biological criteria [22], [34]. In this study as the test set 5 cancer subjects including 3 at early stage (T1N0M0) and 5 controls were taken out from each group .The OPLS-DA models were built up by using the remaining 25 prostate cancer and control samples as a training set using data normalised using the three different normalisation methods and un-normalised data. Figure 2 shows the discrimination of the training set and the prediction of the test set obtained by applying OPLC-DA to the un-normalised data and data normalised using three different methods. Very clear separations between two groups were achieved for the training set in the models generated with data following normalisation to creatinine and MSTUS. Only one cancer and one control sample crossed over the separation line in the model generated with the osmolality method and raw data. The value of R2Y(cum), which explains the discriminative power of the OPLS-DA model, is more than 0.9 with creatinine and MSTUS normalisation and 0.8 with osmolality normalisation but less than 0.7 with un-normalised data. The value of Q2Y(cum), which is calculated by 7-folder cross-validation and able to indicate the predictive power of the model, is about 0.3 for all the methods. Although this value is not as high as expected (>0.5) all the samples in the test set were correctly predicted for all three normalisation methods except one in the MSTUS based model. It is interesting to note that unlike PCA models the OPLS-DA models with different normalisation factors show similar pattern structures and this even the case with non-normalised data. The vectors from the same sample to the other three are quite similar in every OPLS-DA model (Figure 2). Based on the above observations it seems that the normalisation strategy greatly affects the outcome of the unsupervised MVA but not the supervised. However, by removing the deviation of metabolite concentration introduced by urine volume variation, normalisation improves the performance of supervised MVA. The value of Variable Importance for the Projection (VIP) indicates the impact factor of the variable to the model. Generally a variable is regarded important to the model if its VIP value is >1. Overall 406 unique features with VIP values >2 were selected from all three OPLS-DA models. It was found that 76 were common in all three models, 89 were in any two models and 241 were from only one model (Figure S1 in File S4). Those features common in three models showed the highest average VIP values in comparison to the ones common in two or only in one model and they included 80% of top 10 features in VIP value from each model and the remaining 20% could be found in the group which was common in two models. As recommended by Warrack *et al* [27] in order to ensure the reliability of the biomarkers we focused on the VIP features which were common in three normalisation methods. This group included many features which showed the same m/z values under different LC conditions or were detected in both positive and negative ion modes with the same retention time. This observation suggests that these features could be assigned to single metabolites which would make them more reliable as genuine biomarkers. Unpaired Student *t* and ROC tests were performed for these features across all samples and the calculated P-values and area under curve (AUC) obtained by ROC are shown in Table S1 in File S4. As expected all of them showed high AUC (>0.63) and low P-values (<0.05) and their ratios between the cancer and control samples are also very similar each other under different LC-HRMS conditions with different normalisation methods which supports the reliability of the results. Some other features could also be considered as potential biomarkers because of significant statistical results although they were only present in a single LC-HRMS condition. The discriminative ability of sarcosine for prostate cancer was also evaluated (Table S1 in File S3); in keeping with several earlier reports, we found no significant difference in the sarcosine level between cancer and control groups [6], [8]–[10], [12], [15].

### Identification of a potential biomarker panel

**Figure 3:**
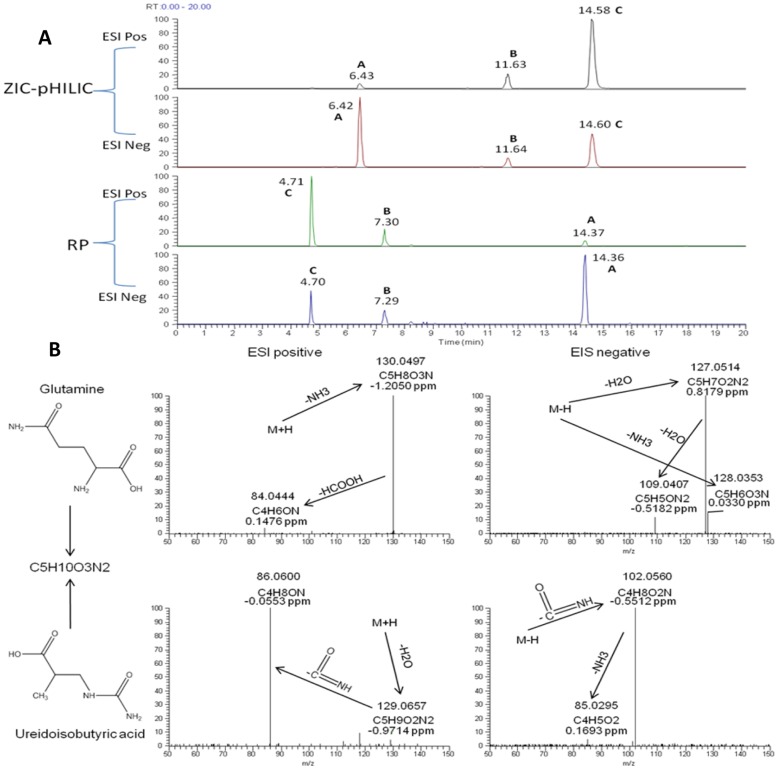
LC-HRMS/MS results of isomers of C_5_H_10_N_2_O_3_. Extracted ion chromatograms under 4 different LC-HRMS conditions (A) and the interpretation of their MS^2^ fragmentations (B).

All the features used in MVA were searched by accurate mass with a 3 ppm tolerance window against an in-house metabolite library [25]. 1673 and 1159 features were putatively identified as metabolites or related signals under ZIC-pHILIC and RP conditions respectively. The identification results in Excel format can be downloaded from our website: http://www.metabolomics.strath.ac.uk and people wishing to file share raw data are welcome to contact us. The biomarkers in File S4 were successfully assigned to genuine metabolites but some of them with several isomers. In order to finalize the identity of the biomarkers an MS^2^ experiment was carried out. A feature mentioned in Table S1 in File S4 (145.062 m/z in ESI negative and 147.076 m/z in ESI positive mode) was assigned the formula C_5_H_10_N_2_O_3_ with mass error less than 1 ppm. Three isomers can be assigned to this formula in the HMDB. These are glutamine, ureido isobutyric acid and alanylglycine. As can been seen in figure 3A the interesting feature refers to Peak B in the extracted ion chromatograms. By MS^2^ analysis Peaks A and C showed the same fragmentation pattern which can be explained as shown in figure 3B and were identified as glutamine by comparing with a standard MS/MS spectrum in the HMDB. Peak B showed a completely different fragmentation pattern and corresponds to ureido isobutyric acid from the interpretation of the MS^2^ spectra. No standard MS/MS spectrum was found in any public database. The fragmentation seems to be directed by the ureido group in the molecule (figure 3B). The absence of a free amine group reduces the polarity of the molecule which explains the opposite elution order to that of glutamine under the two orthogonal LC conditions (figure 3A). Finally by checking the raw data Peak A was identified as being due to an in-source fragment of alpha-N-phenylacetyl-L-glutamine which is a highly abundant component in human urine. Alanylglycine might correspond to the tiny peak before and after Peak B under ZIC-pHILIC and RP conditions respectively. However, its signal at MS level 1 was too weak to obtain reliable MS^2^ fragmentation. The final potential biomarkers and their identities, including MS^2^ fragmentation data, are shown in File S5. More potential biomarkers were identified under the ZIC-pHILIC conditions than under RP conditions. It was interesting to note that there were some biomarkers identified from food e.g. stachydrine and 3-hydroxystachydrine. Their reliability/authenticity as biomarkers was naturally suspected.

From our literature review for cancer diagnosis by metabolomics no studies have assessed the discriminative ability of proposed biomarkers in a single paper by testing samples collected completely independently of the original study. We carried out analysis on urine samples obtained from 30 additional prostate cancer patients and compared data against the control samples analysed earlier. Based on the optimal methodology identified above these 60 samples were compared only under ZIC-pHILIC conditions and the data was normalised against creatinine concentration. In the comparison between the controls and the independently collected prostate cancer samples 14 out of 33 biomarkers were below the 0.05 P-value threshold including ureido isobutyric acid which had almost identical statistical results in both ES1 positive and negative modes (Table 3). Some of the food metabolites did not show significant difference between cancer and healthy groups. Four out of 14 validated biomarkers were identified with confidence as ureido isobutyric acid, indolylacryloyglycine, acetylvanilalinine and 2-oxoglutarate all of which have not been reported in the previous metabolite profiling studies for prostate cancer.

**Table 3:** The statistical results for biomarkers surviving testing against a new cohort of patients.

Polarity	m/z	Rt	Name	Formula	P-value	Ratio
N	268.083	13.47	Unknown^c^	C12H15NO6	<0.0001	4.66
P	236.149	11.51	Unknown^c^	C14H9N2O2	<0.0001	0.37
P	240.102	8.24	N-hydroxy-2-acetamidofluorene^b^	C15H13NO2	0.0003	0.13
N	243.078	6.74	indolylacryloyglycine^a^	C13H12N2O3	0.0007	2.58
**N**	**145.062**	**11.66**	**Ureidoisobutyric acid** a	**C5H10N2O3**	**0.0014**	**0.54**
N	210.026	5.00	Unknown		0.0016	0.30
N	172.025	15.20	Unknown^c^	C6H7NO5	0.0020	2.47
N	252.088	5.01	acetylvanilalinine^a^	C12H15NO5	0.0022	3.94
N	258.992	16.48	Caffeic acid sulfate^a^	C9H8O7S	0.0026	2.29
**P**	**147.077**	**11.66**	**Ureidoisobutyric acid** a	**C5H10N2O3**	**0.0030**	**0.57**
N	401.182	6.40	Thr-Trp-Pro^b^	C20H26N4O5	0.0041	0.49
N	145.014	15.52	2-oxoglutarate^a^	C5H6O5	0.0067	1.97
N	275.023	13.21	Dihydro ferulic acid sulphate^b^	C10H12SO7	0.0332	0.23
P	169.061	7.77	2,3-diaminosalicylic acid^a^	C7H8N2O3	0.0462	2.19

a : identified by accurate mass and MS^2^ fragmentation.

b : only identified by accurate mass in the in-house database.

c : only elemental composition predicted formula (<3 ppm).

**Figure 4:**
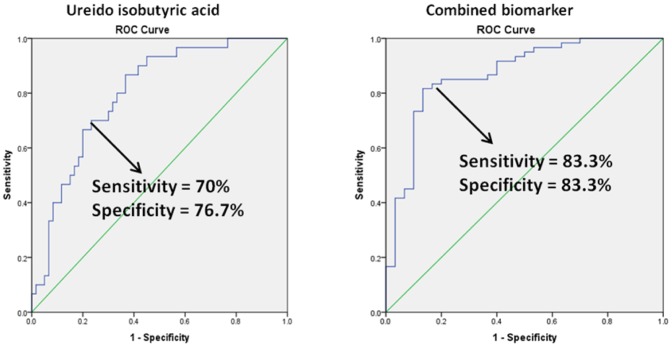
ROC testing results of validated biomarker (UIBA) and the combined biomarker.

Based on the total 90 subjects (60 cancer v.s. 30 controls) the diagnostic power for prostate cancer was evaluated by an ROC test for each of them as well as for their combined power. The peak area was UV scaled and if the level of the biomarker was higher in the cancer group than in the healthy group, it would be treated as positive influence and lower as negative influence. The generated AUC value for the combined biomarkers was 0.896 and the sensitivity and specificity were also improved at the best cut-off point compared to other single biomarkers (Figure 4. The diagnostic efficacy could be improved further by including more single biomarkers but due to their putative identification they were not included in the combination. Overall, the use of the combined biomarker panel is comparable to the use of PSA testing (AUC at 0.94). With future refinement, such a panel may be of clinical use either in isolation or in combination with PSA itself. Due to the limitation of number of subjects measured in this study and the uncertainty of the significance of a few significant biomarkers selected from over a thousand features, further validation of the panel and the specific biomarkers will be performed with more subjects and the data will be publically accessible on our website: http://www.metabolomics.strath.ac.uk.

The four identified biomarkers are not linked in an obvious way although individually they have some interesting biochemical background as biomarkers of other medical conditions. Ureidoisobutyric acid (UIBA) is well established as a urinary marker of an inborn error of metabolism due to ureidopropionase deficiency where its levels are elevated [35]. In the current case its levels are lower in subjects with prostate cancer. UIBA is a metabolite of the DNA base thymidine. UIBA is also a DNA degradation product derived from oxidative damage of thymidine and such damaged bases are removed from DNA by the repair enzyme DNA glycosylase [36]–[38]. Failure to remove such residues can result in mutations being formed during replication due to incorrect base pairing with the damaged site. Lower levels of UIBA might point to reduced rates of excision repair.

Indoylacroylglycine (IAG) has been proposed as a marker for autism in children. Its origin is unclear although it has been proposed that it is produced by metabolic transformation of tryptophan by gut microflora. However, it has been established that its levels fluctuate in human urine according to the time of year and higher levels may relate to increased exposure to UV light. In this respect it is analogous to urocanic acid which is produced from histidine in response to UV radiation [39].

N-acetylvanilalinine (AVA) has been monitored in urine for the detection of a rare inborn error of metabolism due to aromatic amino acid decarboxylase (AAD) deficiency [40]. In the group of biomarkers presented in table 3 there is an unidentified marker (268.083 m/z, negative mode), which like AVA is highly elevated in prostate cancer patients. This marker differs in elemental composition by one oxygen atom from AVA suggesting it might be hydroxylated AVA.

## Conclusions

The current study has established a simple robust protocol for screening of urine for biomarkers by using orthogonal LC methods in conjunction with HRMS. A data extraction protocol based on MZmine was established to remove technical and biologically non-related variations. Applying the developed method we successfully performed a preliminary study on urinary metabolomics for diagnosis of prostate cancer and biomarker discovery. In comparison with previous studies much more comprehensive metabolite profiling was achieved by employing two orthogonal LC methods combined with HRMS which provided a greater opportunity to uncover more potential biomarkers. The lower effect of different normalisation methods on supervised MVA models (OPLS-DA) than on unsupervised (PCA) models was reported for the first time. In addition, a clear improvement in discriminative ability for prostate cancer with normalised data in OPLS-DA models proves the necessity of normalisation. The complete discrimination of men with prostate cancer could be achieved in OPLS-DA models with sensible accuracy of prediction on a test set. The new biomarkers included UIBA which was identified under all LC-HRMS conditions and survived through the validation test of using a new cohort of patients from a different geographic region. The diagnostic ability obtained by combining the validated biomarkers was close to the level of the PSA test. Further validation experiments will be carried in our lab including with more detailed clinical and pathological testing and the testing of further groups of subjects to prove the utility of the biomarkers found in the current study. We hope that other groups pursuing similar protocols aimed at diagnosis of prostate cancer will be able to test the candidate biomarkers we have described

## Supporting Information

File S1 **START checklist for reporting of studies of diagnostic accuracy.**
(DOCX)

File S2 **Text (MZMine 2.10 procedure and settings).**
(DOCX)

File S3 **Text (LC-HRMS results of standard reference compounds), two Figures (representative LC-HRMS chromatograms of alanine, β-alanine and sarcosine under ZIC-pHILIC and C18-AR conditions) and a Table (Student** ***t-test*** **results of scarcosine between cancer and healthy group).**
(DOCX)

File S4 **A Figure (Venn diagram of features with VIP beyond 2 using different normalisation methods) and a Table (statistic data table of some potential biomarkers).**
(DOCX)

File S5 **Text (List of discovered biomarkers with LC-HRMS and MS/MS information under different LC conditions and their statistic data with different normalisation methods).**
(XLSX)
