# Supplementary material for: Application of Holistic Liquid Chromatography-High Resolution Mass Spectrometry Based Urinary Metabolomics for Prostate Cancer Detection and Biomarker Discovery
Source: PLoS One. 2013 Jun 18;8(6):e65880. doi: 10.1371/journal.pone.0065880 (PMC3688815; doi:10.1371/journal.pone.0065880)
Supplement: File S2 — Text (MZMine 2.10 procedure and settings). (DOCX) [file pone.0065880.s002.docx]

MZMine 2.10 procedure and settings

Raw data import

Peak detection

- Mass detection
- Mass detector: Centroid
- Noise level: 1000
- MS level: 1
- Chromatogram builder
- Min time span (min): 0.2
- Min height: 30000
- m/z tolerance: 0.001 m/z or 5 ppm
- Chromatogram Deconvolution: Algorithm: Local minimum search
- Chromatographic threshold: 1%
- Search minimum in RT range (min): 0.4
- Minimum relative height: 5%
- Minimum absolute height: 30000
- Min ratio of peak top/edge: 5
- Peak duration range (min): 0.3-5
- Deisotope
- m/z tolerance: 0.001 m/z or 5 ppm
- Retention time tolerance: 0.1 absolute (min)
- Maximum charge: 2
- Representative isotope: Most intense

Alignment

- Join aligner
- m/z tolerance: 0.001 m/z to 5 ppm
- Weight for m/z: 20
- Retention time tolerance: 5 relative%
- Weight for RT: 10
- Gap filling: Same Rt and m/z range gap filler
- m/z tolerance: 0.001 m/z or 5 ppm
- Filtering: Peak list rows filter
- Minimum peaks in a row: 45
- Minimum peaks in an isotope pattern: 1
- m/z: 75-1000
- Retention time: 3-35
- Peak duration range: 0.2-5

Identification

- Adduct search
- RT tolerance: 0.2 absolute (min)
- Adducts: Na, K, NH4 for ESI positive mode and formate for ESI negative mode and ACN+H for both modes
- m/z tolerance: 0.001 m/z or 5 ppm
- Max relative adduct peak height: 30%
- Complex search
- Ionization method: M+H for ESI positive mode and M-H for negative mode
- Retention time tolerance: 0.2 absolute (min)
- m/z tolerance: 0.001 m/z or 5 ppm
- Max complex peak height: 50%
