# Supplementary material for: Application of Holistic Liquid Chromatography-High Resolution Mass Spectrometry Based Urinary Metabolomics for Prostate Cancer Detection and Biomarker Discovery
Source: PLoS One. 2013 Jun 18;8(6):e65880. doi: 10.1371/journal.pone.0065880 (PMC3688815; doi:10.1371/journal.pone.0065880)
Supplement: File S3 — Text (LC-HRMS results of standard reference compounds), two Figures (representative LC-HRMS chromatograms of alanine, β-alanine and sarcosine under ZIC-pHILIC and C18-AR conditions) and a Table (Student t-test results of scarcosine between cancer and healthy group). (DOCX) [file pone.0065880.s003.docx]

**ZIC-pHILIC-Pos**

| **m/z** | **Rt (min)** | **Name** | **ppm** | **Rt RSD** | **PA RSD** |
| --- | --- | --- | --- | --- | --- |
| 76.03936 | 15.36428 | Glycine | 0.15 | 0.44% | 7.75% |
| 90.05497 | 13.58171 | Sarcosine | 0.15 | 0.40% | 12.07% |
| 90.05497 | 14.90437 | Beta-Alanine | 0.10 | 0.38% | 5.54% |
| 90.05497 | 14.28547 | L-Alanine | 0.07 | 0.56% | 8.33% |
| 104.0706 | 11.81454 | &beta;-alanine-methyl-ester | 0.07 | 0.41% | 4.49% |
| 104.0706 | 13.60979 | DL-3-aminobutyrate | 0.08 | 0.40% | 5.44% |
| 106.0499 | 15.36544 | L-Serine | 0.10 | 0.59% | 15.76% |
| 112.0506 | 10.9376 | Cytosine | 0.11 | 0.29% | 12.22% |
| 114.0663 | 9.355418 | Creatinine | 0.10 | 0.36% | 3.83% |
| 116.0706 | 12.36741 | L-Proline | 0.12 | 0.41% | 3.59% |
| 118.0862 | 10.83397 | Betaine | 0.13 | 0.27% | 4.08% |
| 120.0655 | 14.02336 | L-Threonine | 0.12 | 0.50% | 17.58% |
| 126.022 | 14.56999 | Taurine | 0.08 | 0.44% | 5.03% |
| 127.0502 | 11.51839 | Imidazole-4-acetate | 0.13 | 0.58% | 13.63% |
| 132.0655 | 13.2581 | 5-Aminolevulinate | 0.22 | 0.71% | 15.06% |
| 132.0767 | 14.22783 | creatine | 0.17 | 0.41% | 5.10% |
| 132.1019 | 10.84923 | L-isoleucine | 0.12 | 0.32% | 7.01% |
| 132.1019 | 10.33423 | L-Leucine | 0.15 | 0.33% | 4.28% |
| 137.0462 | 9.88276 | Hypoxanthine | 0.19 | 0.30% | 2.82% |
| 143.0818 | 12.50874 | Ectoine | 0.10 | 0.46% | 25.75% |
| 150.0585 | 11.08554 | L-Methionine | 0.84 | 0.41% | 16.26% |
| 150.1126 | 8.505976 | Triethanolamine | 0.33 | 0.18% | 7.12% |
| 152.0566 | 11.94474 | Guanine | 0.38 | 0.34% | 3.92% |
| 152.0708 | 6.627482 | 4-Hydroxyphenylacetaldoxime | 0.16 | 1.20% | 23.24% |
| 156.0769 | 14.56926 | L-Histidine | 0.06 | 0.67% | 10.01% |
| 166.0863 | 9.679388 | L-Phenylalanine | 0.11 | 0.43% | 3.89% |
| 170.0925 | 12.59437 | N(pi)-Methyl-L-histidine | 0.10 | 0.59% | 3.02% |
| 175.1189 | 25.25512 | L-Arginine | 0.03 | 0.66% | 22.38% |
| 180.0865 | 14.04321 | D-Glucosamine | 0.25 | 0.49% | 8.92% |
| 189.1233 | 14.62051 | N6-Acetyl-L-Lysine | 0.15 | 0.46% | 3.82% |
| 190.0709 | 14.00443 | N-Acetyl-L-glutamate | 0.13 | 0.55% | 11.86% |
| 192.0654 | 13.22356 | 5-Hydroxyindoleacetate | 0.18 | 0.44% | 6.69% |
| 204.1228 | 10.80518 | O-Acetylcarnitine | 0.23 | 0.32% | 11.53% |
| 205.097 | 11.20638 | L-Tryptophan | 0.21 | 0.29% | 2.10% |
| 209.0918 | 10.3898 | L-Kynurenine | 0.26 | 0.38% | 6.34% |
| 220.1183 | 8.427013 | Pantothenate | 2.43 | 0.62% | 5.03% |
| 222.0969 | 11.34349 | N-Acetyl-D-Glucosamine | 0.04 | 0.45% | 7.02% |
| 244.0923 | 11.48813 | Cytidine | 0.18 | 0.38% | 5.86% |
| 268.1038 | 8.591168 | Adenosine | 0.26 | 0.43% | 5.85% |
| 285.0826 | 12.16616 | Xanthosine | 0.23 | 0.52% | 6.93% |
| 298.0964 | 6.470457 | 5'-Methylthioadenosine | 0.17 | 1.68% | 22.00% |
| 377.1454 | 8.104085 | Riboflavin | 0.26 | 0.49% | 26.65% |

**ZIC-pHLIC-Neg**

| 88.04027 | 14.3686 | Beta-Alanine | 0.26 | 0.43% | 12.55% |
| --- | --- | --- | --- | --- | --- |
| 102.056 | 13.71926 | DL-3-aminobutyrate | 0.35 | 0.39% | 15.13% |
| 102.056 | 11.80259 | &beta;-alanine-methyl-ester | 0.20 | 0.41% | 19.51% |
| 103.004 | 15.71506 | Malonate | 0.34 | 0.55% | 8.98% |
| 103.0401 | 9.066948 | (R)-3-Hydroxybutanoate | 0.12 | 0.67% | 7.58% |
| 103.0401 | 7.897369 | 2-Hydroxybutanoic acid | 0.11 | 0.86% | 6.21% |
| 104.0352 | 15.37994 | L-Serine | 0.28 | 0.53% | 9.64% |
| 112.0517 | 9.362606 | Creatinine | 0.11 | 0.39% | 8.42% |
| 114.056 | 12.40021 | L-Proline | 0.21 | 0.47% | 27.12% |
| 115.0036 | 15.96825 | Fumarate | 0.08 | 0.51% | 44.49% |
| 115.0037 | 12.78486 | Maleic acid | 0.40 | 0.86% | 5.47% |
| 116.0717 | 10.86311 | Betaine | 0.16 | 0.41% | 7.44% |
| 116.0717 | 12.09013 | L-Valine | 0.19 | 0.53% | 7.16% |
| 118.051 | 14.10821 | L-Homoserine | 0.21 | 0.57% | 12.66% |
| 124.0074 | 14.56098 | Taurine | 0.35 | 0.44% | 3.01% |
| 125.0357 | 11.52326 | Imidazole-4-acetate | 0.23 | 0.56% | 25.63% |
| 128.0354 | 10.09917 | 5-Oxoproline | 0.26 | 0.48% | 5.23% |
| 130.0623 | 14.25921 | creatine | 0.26 | 0.41% | 4.41% |
| 130.0874 | 10.39815 | L-isoleucine | 0.07 | 0.23% | 6.03% |
| 135.0314 | 9.871064 | Hypoxanthine | 0.17 | 0.30% | 2.52% |
| 141.067 | 12.66086 | Ectoine | 0.30 | 0.60% | 121.55% |
| 145.0143 | 15.51866 | 2-Oxoglutarate | 0.38 | 0.51% | 16.02% |
| 146.046 | 10.56258 | L-Glutamate | 0.12 | 0.54% | 16.90% |
| 147.03 | 15.10539 | Citramalate | 0.26 | 0.66% | 6.22% |
| 148.0438 | 11.09119 | L-Methionine | 0.29 | 0.29% | 9.12% |
| 149.0456 | 12.64355 | L-Arabinose | 0.25 | 0.57% | 6.62% |
| 150.0562 | 6.569857 | 4-Hydroxyphenylacetaldoxime | 0.19 | 1.57% | 18.25% |
| 151.0263 | 10.33888 | Alloxanthine | 0.12 | 0.34% | 9.01% |
| 154.0623 | 14.43556 | L-Histidine | 0.30 | 0.93% | 10.88% |
| 157.0368 | 13.51501 | Allantoin | 0.15 | 0.25% | 3.65% |
| 164.0718 | 9.685774 | L-Phenylalanine | 0.18 | 0.38% | 8.29% |
| 167.035 | 8.864493 | Homogentisate | 0.40 | 0.57% | 8.57% |
| 168.0779 | 12.49896 | N(pi)-Methyl-L-histidine | 0.19 | 0.60% | 7.99% |
| 174.0409 | 14.49041 | N-Acetyl-L-aspartate | 0.28 | 0.54% | 13.72% |
| 179.0564 | 12.97176 | D-Fructose | 0.16 | 0.85% | 6.51% |
| 181.0719 | 13.61714 | D-Sorbitol | 0.20 | 0.40% | 8.38% |
| 187.1088 | 14.56393 | N6-Acetyl-L-Lysine | 0.45 | 0.38% | 3.90% |
| 188.0565 | 13.99503 | N-Acetyl-L-glutamate | 0.21 | 0.58% | 16.63% |
| 190.051 | 13.22305 | 5-Hydroxyindoleacetate | 0.23 | 0.34% | 8.80% |
| 193.0354 | 15.98395 | D-Galacturonate | 0.27 | 0.52% | 14.12% |
| 202.1086 | 10.57964 | O-Acetylcarnitine | 0.21 | 0.34% | 7.92% |
| 203.0827 | 11.22636 | L-Tryptophan | 0.21 | 0.29% | 4.92% |
| 207.0775 | 10.41229 | L-Kynurenine | 0.32 | 0.12% | 43.13% |
| 218.1036 | 8.448383 | Pantothenate | 0.27 | 0.62% | 5.90% |
| 220.0827 | 11.788 | N-Acetyl-D-Glucosamine | 0.26 | 0.69% | 6.76% |
| 242.0785 | 11.49979 | Cytidine | 0.25 | 0.56% | 19.47% |
| 266.0898 | 8.611374 | Adenosine | 0.37 | 0.33% | 5.83% |
| 267.0739 | 10.46604 | Inosine | 0.41 | 0.38% | 90.46% |
| 283.0686 | 12.16324 | Xanthosine | 0.25 | 0.52% | 2.16% |
| 341.1092 | 15.8559 | Maltose | 0.46 | 0.44% | 98.17% |
| 341.1093 | 14.69338 | Sucrose | 0.28 | 0.40% | 6.66% |
|  |  |  |  |  |  |

**C18-AR-Pos**

| 76.03936 | 4.678633 | Glycine | 0.11 | 0.11% | 12.07% |
| --- | --- | --- | --- | --- | --- |
| 90.05498 | 4.904009 | Beta-Alanine | 0.14 | 0.35% | 9.64% |
| 104.0706 | 5.233273 | &beta;-alanine-methyl-ester | 0.10 | 0.11% | 10.79% |
| 106.0498 | 4.830747 | L-Serine | 0.22 | 0.19% | 7.12% |
| 114.0662 | 5.278657 | Creatinine | 0.13 | 0.40% | 11.51% |
| 118.0862 | 5.529659 | L-Valine | 0.17 | 0.22% | 5.84% |
| 118.0862 | 5.129682 | Betaine | 0.21 | 0.32% | 5.86% |
| 122.0963 | 12.04347 | 1-Phenylethylamine | 0.42 | 0.21% | 44.18% |
| 123.0551 | 6.965282 | Nicotinamide | 0.21 | 0.39% | 35.64% |
| 126.0219 | 4.893701 | Taurine | 0.13 | 0.31% | 8.27% |
| 130.0497 | 7.785421 | 5-Oxoproline | 0.08 | 0.26% | 9.70% |
| 132.0766 | 5.155742 | creatine | 0.17 | 0.22% | 9.11% |
| 132.1017 | 8.330041 | L-Leucine | 0.19 | 0.20% | 9.48% |
| 133.097 | 4.463601 | L-Ornithine | 0.29 | 0.12% | 9.29% |
| 136.112 | 12.65896 | Amphetamine | 0.48 | 0.18% | 8.30% |
| 137.0458 | 7.146662 | Hypoxanthine | 0.22 | 0.09% | 5.09% |
| 146.1651 | 3.865108 | Spermidine | 0.28 | 1.87% | 12.41% |
| 147.1128 | 4.453059 | L-Lysine | 0.16 | 0.25% | 5.00% |
| 150.0582 | 7.102887 | L-Methionine | 0.14 | 0.21% | 3.72% |
| 153.0406 | 7.845913 | Alloxanthine | 0.21 | 0.32% | 15.14% |
| 154.0861 | 7.306986 | Dopamine | 0.22 | 0.44% | 10.51% |
| 156.0768 | 4.478383 | L-Histidine | 0.07 | 0.12% | 5.63% |
| 166.0861 | 10.38773 | L-Phenylalanine | 0.09 | 0.18% | 7.11% |
| 169.0354 | 7.100971 | Urate | 0.26 | 0.19% | 11.10% |
| 176.0552 | 6.936115 | N-Acetyl-L-aspartate | 0.20 | 1.46% | 56.45% |
| 180.0864 | 4.819876 | D-Glucosamine | 0.44 | 0.25% | 24.24% |
| 183.0863 | 4.953169 | Mannitol | 0.34 | 0.11% | 5.28% |
| 190.0708 | 7.689826 | N-Acetyl-L-glutamate | 0.12 | 0.35% | 9.63% |
| 192.0653 | 14.64764 | 5-Hydroxyindoleacetate | 0.27 | 0.11% | 10.40% |
| 193.0341 | 6.987561 | Citrate | 0.19 | 2.14% | 12.87% |
| 205.0971 | 12.4993 | L-Tryptophan | 0.26 | 0.19% | 7.38% |
| 209.0918 | 11.09384 | L-Kynurenine | 0.28 | 0.13% | 11.14% |
| 220.1178 | 10.18595 | Pantothenate | 0.18 | 0.10% | 12.11% |
| 222.0969 | 5.328555 | N-Acetyl-D-Glucosamine | 1.81 | 0.26% | 29.60% |
| 268.1037 | 8.217839 | Adenosine | 0.24 | 0.45% | 6.72% |
| 269.0877 | 8.804627 | Inosine | 0.30 | 1.87% | 44.28% |
| 285.0826 | 9.261984 | Xanthosine | 0.18 | 0.25% | 6.07% |
| 343.1232 | 4.92345 | Sucrose | 0.50 | 0.44% | 8.45% |
| 377.1454 | 13.93333 | Riboflavin | 0.30 | 0.13% | 8.71% |

**C18-AR-Neg**

| 103.0036 | 6.194729 | Malonate | 0.17 | 0.26% | 26.12% |
| --- | --- | --- | --- | --- | --- |
| 103.0401 | 8.739526 | 2-Hydroxybutanoic acid | 0.12 | 0.12% | 11.96% |
| 112.0517 | 5.296733 | Creatinine | 0.19 | 0.95% | 20.21% |
| 115.0037 | 7.776559 | Fumarate | 0.13 | 0.64% | 7.84% |
| 117.0194 | 8.461357 | Methylmalonate | 0.28 | 0.32% | 22.50% |
| 124.0075 | 4.884335 | Taurine | 0.22 | 0.36% | 3.80% |
| 128.0354 | 7.709135 | 5-Oxoproline | 0.16 | 0.28% | 11.07% |
| 130.0623 | 5.191067 | creatine | 0.24 | 0.22% | 17.27% |
| 133.0143 | 5.824973 | (R)-Malate | 0.15 | 0.31% | 11.73% |
| 146.046 | 5.560285 | L-Glutamate | 0.24 | 0.10% | 8.08% |
| 147.03 | 7.906362 | Citramalate | 0.17 | 0.40% | 15.32% |
| 149.0456 | 8.710483 | D-Xylose | 0.21 | 0.10% | 12.45% |
| 149.0457 | 5.319193 | L-Arabinose | 0.18 | 0.10% | 5.02% |
| 150.0562 | 10.66666 | 4-Hydroxyphenylacetaldoxime | 0.19 | 0.24% | 13.65% |
| 151.0263 | 7.857283 | Alloxanthine | 0.17 | 0.38% | 9.48% |
| 154.0623 | 4.693913 | L-Histidine | 0.22 | 0.48% | 8.08% |
| 156.9966 | 10.35118 | Benzenesulfonate | 0.30 | 0.14% | 15.20% |
| 157.0368 | 5.325953 | Allantoin | 0.13 | 0.31% | 9.00% |
| 163.0614 | 5.635189 | &beta;-L-fucose | 0.17 | 0.17% | 11.41% |
| 164.0719 | 10.39684 | L-Phenylalanine | 0.52 | 0.18% | 23.58% |
| 165.0194 | 14.88706 | Phthalate | 0.14 | 0.17% | 25.74% |
| 167.0212 | 7.096227 | Urate | 0.24 | 0.21% | 10.31% |
| 168.078 | 4.763835 | N(pi)-Methyl-L-histidine | 0.20 | 0.11% | 7.54% |
| 177.0407 | 5.220268 | D-Galactono-1,4-lactone | 0.17 | 0.25% | 6.08% |
| 179.0563 | 5.221793 | D-Fructose | 0.41 | 0.34% | 5.98% |
| 188.0566 | 7.701217 | N-Acetyl-L-glutamate | 0.27 | 0.35% | 17.74% |
| 191.02 | 7.003265 | Citrate | 0.16 | 0.09% | 5.89% |
| 193.0355 | 4.895782 | D-Galacturonate | 0.19 | 0.32% | 6.49% |
| 195.0512 | 4.985 | D-Gluconic acid | 0.21 | 0.23% | 4.44% |
| 218.1036 | 10.18355 | Pantothenate | 0.19 | 0.09% | 11.03% |
| 220.0828 | 5.211284 | N-Acetyl-D-Glucosamine | 0.40 | 0.32% | 9.05% |
| 267.0737 | 8.719255 | Inosine | 0.21 | 2.31% | 62.04% |
| 283.0687 | 9.259603 | Xanthosine | 0.22 | 0.24% | 6.60% |
| 341.1094 | 5.274197 | Sucrose | 0.42 | 0.27% | 5.69% |

Figure.1 Clear separation of sarcosine from its isomers alanine and β-alanine in urine under ZIC-pHILIC ESI positive condition can be confirmed by comparison of the retention times to their standards.


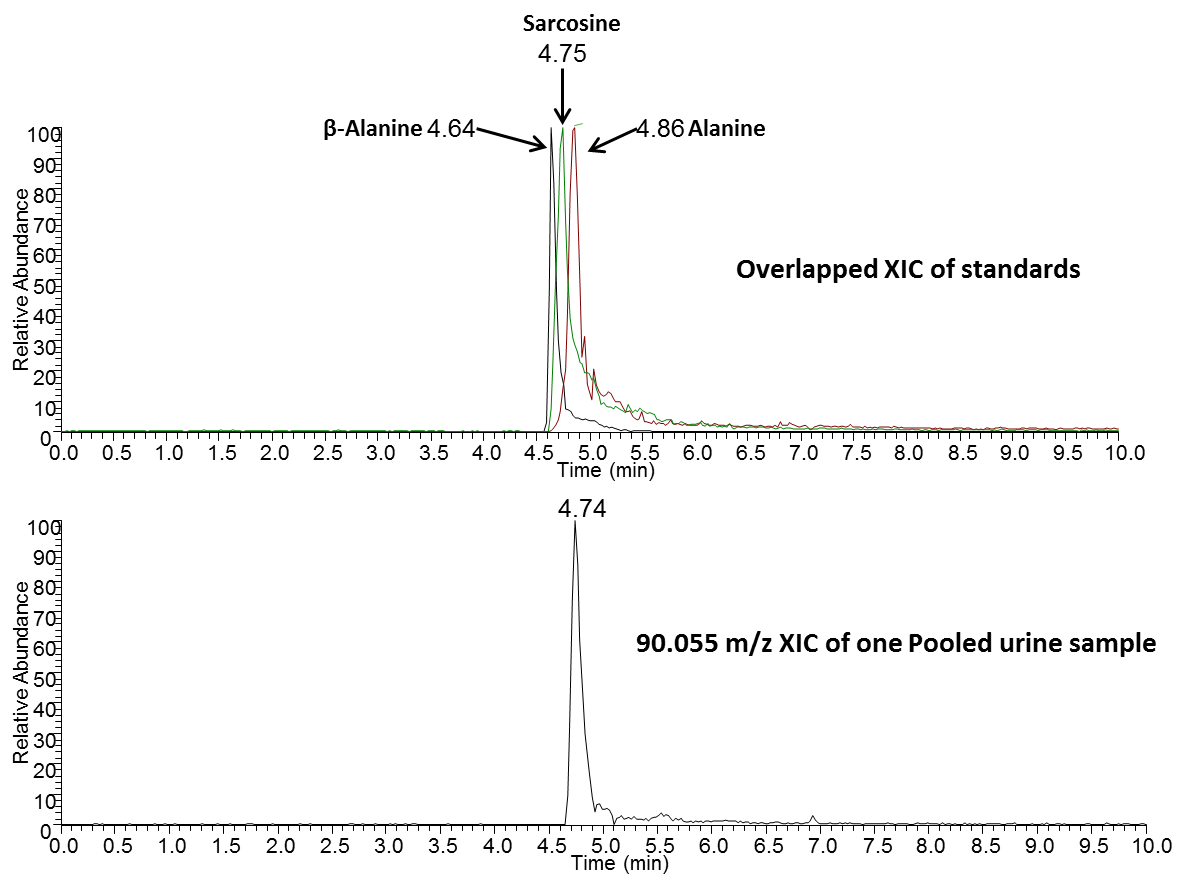


Figure.2 Sarcosine elutes at dead volume without separation from its isomers alanine and β-alanine under RP ESI positive condition. Only one peak can be observed in a real pooled urine sample.

| Normalization | P-value | Ratio |
| --- | --- | --- |
| MSTUS | 0.4583 | 0.86 |
| Creatinine | 0.1556 | 0.64 |
| Osmolality | 0.2045 | 0.76 |
| Alanine | 0.1301 | 0.82 |

Table.1 No significant difference can be shown on scarcosine between cancer and healthy groups with any normalization method.
