# Supplementary material for: Application of Holistic Liquid Chromatography-High Resolution Mass Spectrometry Based Urinary Metabolomics for Prostate Cancer Detection and Biomarker Discovery
Source: PLoS One. 2013 Jun 18;8(6):e65880. doi: 10.1371/journal.pone.0065880 (PMC3688815; doi:10.1371/journal.pone.0065880)
Supplement: File S4 — A Figure (Venn diagram of features with VIP beyond 2 using different normalisation methods) and a Table (statistic data table of some potential biomarkers). (DOCX) [file pone.0065880.s004.docx]

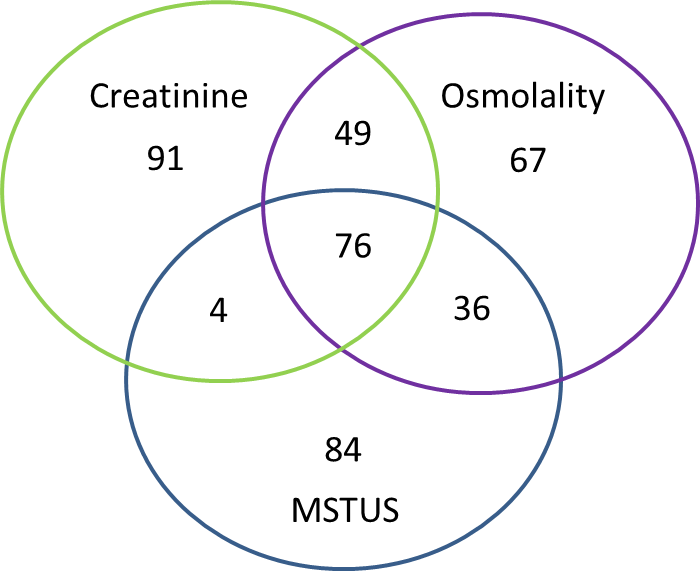


Figure.1 With VIP value beyond 2, 76 features are common in three normalisation methods, creatinine and osmolality share 49 pairs of features, creatinine and MSTUS only share 4 pairs, Osmolality and MSTUS share 36 pairs.

| LC-MS | m/z | Rt | AUC | | | P-value | | | Ratio | | | RSD% |
| --- | --- | --- | --- | --- | --- | --- | --- | --- | --- | --- | --- | --- |
|  |  |  | **C** | **M** | **O** | **C** | **M** | **O** | **C** | **M** | **O** |  |
| ZIC-pHILIC-P | 130.086 | 10.68 | 0.814 | 0.778 | 0.777 | 0.0008 | 0.0006 | 0.0012 | 0.24 | 0.26 | 0.22 | 4.14 |
| RP-P |  | 5.55 | 0.783 | 0.728 | 0.724 | 0.0059 | 0.0047 | 0.0049 | 0.44 | 0.5 | 0.44 | 7.49 |
| ZIC-pHILIC-P | 144.102 | 10.28 | 0.781 | 0.746 | 0.743 | 0.0021 | 0.002318 | 0.00553 | 0.38 | 0.47 | 0.4 | 6.97 |
| RP-P |  | 5.62 | 0.796 | 0.739 | 0.758 | 0.0007 | 0.0031 | 0.0033 | 0.38 | 0.49 | 0.41 | 6.8 |
| RP-N | 145.062 | 7.49 | 0.792 | 0.74 | 0.724 | 0.0002 | 0.0042 | 0.0188 | 0.54 | 0.67 | 0.75 | 8.97 |
| ZIC-pHILIC-N |  | 11.61 | 0.794 | 0.77 | 0.739 | 0.0012 | 0.009 | 0.0035 | 0.56 | 0.72 | 0.68 | 5.93 |
| RP-P | 147.076 | 7.5 | 0.866 | 0.758 | 0.762 | <0.0001 | 0.0006 | 0.0002 | 0.54 | 0.71 | 0.68 | 7.87 |
| ZIC-pHILIC-P |  | 11.6 | 0.744 | 0.764 | 0.69 | 0.004 | 0.0118 | 0.0162 | 0.61 | 0.78 | 0.72 | 5.62 |
| ZIC-pHILIC-P | 160.097 | 11.86 | 0.772 | 0.74 | 0.747 | 0.0019 | 0.0052 | 0.0031 | 0.3 | 0.39 | 0.3 | 3.42 |
| RP-P |  | 5.21 | 0.811 | 0.746 | 0.754 | 0.0014 | 0.0024 | 0.0025 | 0.26 | 0.32 | 0.26 | 7.22 |
| ZIC-pHILIC-N | 243.078 | 6.23 | 0.748 | 0.741 | 0.716 | 0.0013 | 0.0027 | 0.0095 | 0.38 | 0.36 | 0.38 | 5.72 |
| RP-N |  | 18.57 | 0.71 | 0.659 | 0.668 | 0.005 | 0.0183 | 0.0411 | 0.49 | 0.56 | 0.57 | 4.7 |
| RP-N | 245.093 | 18.82 | 0.687 | 0.648 | 0.648 | 0.0049 | 0.0077 | 0.0148 | 0.43 | 0.49 | 0.48 | 9.37 |
| RP-P | 247.107 |  | 0.682 | 0.632 | 0.633 | 0.0059 | 0.0238 | 0.0335 | 0.41 | 0.49 | 0.48 | 4.5 |

Table.1 C=Creatinine M=MSTUS O=Osmolality Ratio=mean of cancer/mean of healthy RSD%=relative standard deviation of the feature in QCs under this LC-MS condition
